# Providing Value to New Health Technology: The Early Contribution of Entrepreneurs, Investors, and Regulatory Agencies

**DOI:** 10.15171/ijhpm.2017.11

**Published:** 2017-01-25

**Authors:** Pascale Lehoux, Fiona A. Miller, Geneviève Daudelin, Jean-Louis Denis

**Affiliations:** ^1^ Department of Health Management, Evaluation and Policy, School of Public Health, University of Montreal, Montréal, QC, Canada.; ^2^ Institute of Public Health Research of University of Montreal (IRSPUM), Montréal, QC, Canada.; ^3^ University of Montreal Chair on Responsible Innovation in Health Montreal, Montréal, QC, Canada.; ^4^ Institute of Health Policy, Management and Evaluation, University of Toronto, Toronto, ON, Canada.; ^5^ École Nationale d’administration publique (ENAP), Quebec City, QC, Canada.; ^6^ Department of Management, Faculty of Social Science & Public Policy, King’s College, London, UK.

**Keywords:** Innovation Policy, Health Policy, Health Technology Development (HTA), Technology-Based Ventures, Early HTA

## Abstract

**Background:** New technologies constitute an important cost-driver in healthcare, but the dynamics that lead to their emergence remains poorly understood from a health policy standpoint. The goal of this paper is to clarify how entrepreneurs, investors, and regulatory agencies influence the value of emerging health technologies.

**Methods:** Our 5-year qualitative research program examined the processes through which new health technologies were envisioned, financed, developed and commercialized by entrepreneurial clinical teams operating in Quebec’s (Canada) publicly funded healthcare system.

**Results:** Entrepreneurs have a direct influence over a new technology’s value proposition, but investors actively transform this value. Investors support a technology that can find a market, no matter its intrinsic value for clinical practice or healthcare systems. Regulatory agencies reinforce the "double" value of a new technology—as a health intervention and as an economic commodity—and provide economic worth to the venture that is bringing the technology to market.

**Conclusion:** Policy-oriented initiatives such as early health technology assessment (HTA) and coverage with evidence may provide technology developers with useful input regarding the decisions they make at an early stage. But to foster technologies that bring more value to healthcare systems, policy-makers must actively support the consideration of health policy issues in innovation policy.

## Background

### Technological Innovation in Healthcare

Health services and policy scholars frequently underscore that technology constitutes one important cost-driver that needs to be better managed, but less often fully acknowledge that a technological solution can be designed in many different ways.^1-3^ Researchers focus their attention downstream, after the adoption of a new technology and develop “little insight” into the processes that led to its existence.^4^ Yet, the upstream dynamics that affect the emergence of technologies have their own ways of picking “winners” up and tossing “losers” aside, which may not align with health policy priorities.^5^ This is why policy-oriented initiatives such as early health technology assessment (HTA) and “coverage with evidence development” are gaining traction, seeking to inform innovation at an earlier stage.^6-9^

The goal of this paper is to contribute to this body of policy-oriented research by clarifying how entrepreneurs, investors, and regulatory agencies define and influence the value of a new technology at an early stage, which spans for a medical device from 5 to 10 years of development.^10^ Our qualitative fieldwork was structured around five ventures that had been created by clinical teams in the mid 1990s in Quebec (Canada), had secured capital investment and obtained regulatory approval for their core technology. We interviewed those who were part of the design team as well as policy-makers, investors, and regulatory experts. We also analyzed documents that described the evolution of the ventures since their inception.

Below, we introduce a framework of the supply- and demand-side logics that affect health technology development and summarize our methodology. Then, our findings examine the respective contribution of entrepreneurs, investors, and regulatory agencies to a new technology’s value proposition.^11^ Our discussion underscores that early HTA and coverage with evidence development initiatives could provide technology developers with useful input regarding the decisions they make. Yet, to encourage the creation of technologies that bring more value to healthcare systems, a more comprehensive health innovation policy is needed.

### The Interplay Between Health Innovation Supply and Demand

Since the late 1980s, health policy-makers in most industrialized countries have been concerned with the adoption and diffusion of new medical technology.^12-14^ They relied among other policy tools on HTA, a process that takes place once the technology is on the market and clinical evidence has been generated. Although HTA has gained recognition worldwide, the decisions made on each and every new technology by policy-makers, healthcare managers, clinicians, and patients remain multidimensional and the evidence needed often arrives late in the adoption process.^5^ Recently, the HTA community has increased its efforts to produce evidence-based recommendations sooner through early HTA. According to the systematic review of Markiewicz et al,^7^ there is great diversity in the early HTA methods currently used:

“Early assessment comprises a strategic analysis (including stakeholders analysis) of the medical context and the competition, evaluation of the economic impact of medical devices and early assessment of clinical effectiveness of the medical devices under development, all with the aim to reduce uncertainty in the developmental stage of a medical device.”

The HTA community has also begun examining technologies for which disinvestment would be appropriate, hoping that valuable innovations could be funded through budget reallocation.^6^ Third-party payers in the United States are, indeed, backing off from a “largely cost-unconscious demand.”^15^ To provide “promising new technologies” while gathering further evidence about their effectiveness, coverage with evidence development strategies have been deployed in North America and Europe.^8^ These strategies consist in a pre-market assessment of a technology’s clinical utility, safety, and efficacy in view of licensing and coverage criteria.^8^ The objectives are to generate regulatory evidence at an earlier stage, decrease risks for manufacturers and investors, and thus, encourage and expedite the innovation process.

The notion that the value of new technology should be defined in terms of *health policy outcomes* (eg, patient safety, quality-adjusted life years, cost-savings, etc) stands in contrast with innovation policy frameworks where technological innovation is viewed as a means to generate wealth.^16-18^ Innovation policy relies on a combination of fiscal arrangements and research & development (R&D) funding tools. For instance, Horizon 2020 projected to spend up to EUR 353.75 million in 2016 to support innovation by small and medium sized enterprises (SMEs). The aim of the SME policy instrument is to assist “innovative SMEs to shape new markets, create growth, and achieve high return on investment.”^19^ The value of technological innovation is here defined in terms of *innovation policy outcomes*, eg, new ventures, highly qualified jobs, profits, etc. One may, thus, wonder about the way the interplay between innovation policy and health policy, which value different outcomes and operate according to different logics, affects the development of new health technologies.^20^ This is the research question this paper seeks to address.

The framework depicted in Figure derives from the literature and draws on our previous phases of fieldwork research.^10,21,22^ This framework does not do full justice to the nuances and variations that characterize specific health innovation policies across industrialised countries.^2,3^ But it illustrates that innovation policy contributes to supply-side dynamics and health policy to demand-side dynamics with very few coordination mechanisms to align their respective actions.^5^ Typically, innovation policies and health policies are governed as 2 distinct government portfolios, which draw their respective directions and resources from 2 different ministries.^6^

**Figure:**
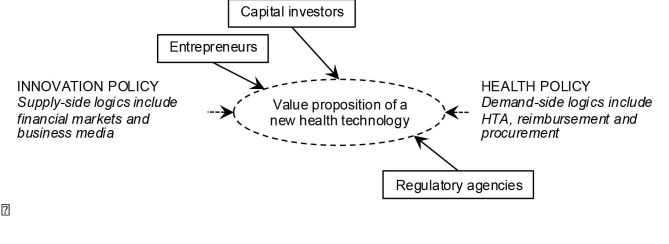
Analytical Framework.

The framework suggests, more specifically, that entrepreneurs, investors, and regulatory agencies may influence the *value proposition* of a new technology, which refers to “the value created for users by the offering based on the technology.”^11^ Although clinicians who create ventures may have a sense of users’ expectations, their activities are conditioned by supply-side logics, including financial markets and business media (which encompasses electronic and print newspapers and magazines that cover business and economic issues). Capital investors define the level and types of financial and human resources made available for a venture to engage in R&D activities and develop a business plan.^21^ As emerging ventures must convince shareholders and business analysts that through successive advances they are getting closer to the goal of generating returns on investment, financial markets and the business-oriented media are influential since they affect the level of enthusiasm and trust investors may manifest toward specific ventures.^16,17^ Among the demand-side logics, health policy concerns become more tangible once innovations have obtained regulatory approval and are introduced into clinical practice. Since ventures seek to penetrate several markets simultaneously or successively, this framework acknowledges that those who steer their development may take regulatory, HTA, reimbursement and procurement strategies into consideration.^3,13^

## Methods

To generate an in-depth analysis of how key innovation stakeholders contribute to technology development, our fieldwork involved a phased approach wherein we gathered a multifaceted corpus of qualitative data. According to Sutton,^22^ extensive and carefully reported qualitative fieldwork provides a legitimate and important contribution to the management scholarship by emphasizing meanings, logics, and processes.

Our data collection strategy was organized around 5 Montreal-based ventures that had emerged from healthcare settings in the mid 1990s and whose core innovations were in the early stage of commercialization when our study began in 2008. We purposefully selected cases whose “information content” was likely to be rich and accessible.^23^ These ventures had all won awards for entrepreneurship and Montreal, the largest metropolitan centre in Quebec, had established a solid presence in the Canadian medical device industry. By choosing ventures that had started their R&D operations at the same period and in the same province, we, thus, included cases that evolved within a similar policy context and economic climate.

More specifically, Table 1 indicates the gaps the 5 ventures sought to address in the domains of breast imaging, cardiology, obstetrics, home monitoring and orthopaedic surgery and the value propositions of their technology. These cases offered variations along our analytical constructs,^23^ a feature likely to increase the theoretical relevance of our observations. The technologies they developed perform different clinical functions — diagnostic, therapeutic, decision-support, and monitoring — and were targeted at different users and purchasers. As a result, these ventures’ technology development activities involved overcoming different technical and clinical challenges and negotiating the expectations of different stakeholders and users.

**Table 1:** The Gap Addressed by 5 Health Technology-Based Ventures and Their Technology’s Value Proposition

**Innovation**	**Gap**	**Value Proposition**
Optical molecular imaging device for breast cancer diagnosis and characterisation	Limitations and risks in mammography	Providing early breast cancer diagnostic and eventually monitoring treatment more safely
Line of cryoablation catheters for the treatment of arrhythmia	Risks of existing heat-based treatments	Providing an improved technology that may cure a widespread health problem
Decision-support software to monitor prolonged labour and abnormal foetal heart rates and help detect birth-related injuries	Subjectivity underlying obstetrical practice	Preventing rare but costly obstetrical complications by predicting objectively birth-related injuries
Home telehealth solution comprising a set of coordination tools to promote continuity of care for chronically ill patients	Poor management of chronic illness and uncoordinated services	Improving the management of chronically ill patients and preventing the use of costly resources through home monitoring and patient empowerment
Computer-assisted navigation system to support MIS orthopaedic surgery such as hip, knee, and spine implants	Limitations and risks in orthopaedic MIS	Increasing accuracy in orthopaedic surgical practice and reducing complications

Abbreviation: MIS, minimally invasive.

As Table 2 summarizes, we gathered multiple data sources during the preliminary data collection phase, the detailed data collection phase and the analysis and debriefing phase of our fieldwork. Qualitative data included exploratory interviews, which helped our team to get a better sense of the financial, commercial and regulatory challenges affecting technology development and to subsequently refine our data collection tools and strategies. We also conducted formal interviews that provided us with a significant understanding of how external stakeholders interact with health technology ventures. All interviews were recorded and transcribed verbatim (transcriptions were sent to respondents for validation). The content of the ventures’ websites was retrieved between October and December 2008 and an extensive media coverage analysis was performed (1998-2009). All these downloadable documents were indexed and analyzed, including press releases and annual reports.

**Table 2:** The Data Gathered During Our Fieldwork

**Fieldwork Component**	**Data Sources**
Preliminary data collection phase: • To generate an overview of the phenomenon, to gather key information about 5 ventures, to select the three cases to be documented in the subsequent phase and adjust data collection tools accordingly	• Exploratory interviews (60-120 min) with CEOs and high-level executives of 5 ventures and with experts in regulatory affairs and technology transfer (n = 11)
	• Analysis of all documents retrieved in 2008 that described the activities of the ventures since their inception (mid 1990s): • Press releases (n = 568) • Annual reports (n = 21) • Promotional documents (n = 23)
Detailed data collection phase: • To generate an in-depth understanding of technology developers’ practices and rationales	• Semi-structured interviews (90-120 min) with clinicians and scientists who contributed to the creation of three ventures (n = 9)
• To document external stakeholders’ practices and contribution to technology development processes	• Semi-structured interviews (35-120 min) with capital investors (n = 6), regulators (n = 3), and policy-makers (n = 5)
	• Notes recorded during and after industry events observation (n = 6)
	• Media coverage analysis of all printed and electronic content published in English or French (n = 814) that mentioned the ventures, their CEOs and products between 1998 and 2009, retrieved through CBCA and *Biblio Branchée* databases
Analysis and debriefing phase: • To analyse and share findings, obtain feedback and collect additional data	• Scientific and policy-oriented presentations of preliminary findings (n = 14)
	• Three mixed focus groups that engaged in two structured deliberations (60 min each with technology developers, clinicians, and patient representatives (n = 19)

Abbreviation: CEO, chief executive officer.

Consistent with a phased fieldwork approach, we adopted an iterative analytical strategy in which our theorizing efforts alternated with empirical analyses.^22^ Our analyses were supported by the QDA Miner qualitative data analysis software, which enabled us to constitute an integrated database. To support the different analyses that we had planned for our fieldwork, we applied a mixed coding strategy wherein codes were both deductively and inductively generated and regularly discussed within the research team. For the current paper, the analytical aim we pursued was to flesh out the overarching lessons of our research program with the help of the framework presented earlier.^23^ To reconstruct with as much accuracy as possible the processes that interviewees described, the interviews’ content was triangulated with the other data sources. To this end, we extracted factual information from documents that covered on average a 15-year period. Applying a constant comparative method of analysis, our team actively searched for evidence that would contradict our emerging analytical insights. Finally, during the analysis and debriefing phase, we gathered key feedback from technology developers, clinicians, and patient representatives.

In the next section, we clarify how entrepreneurs, investors, and regulatory agencies respectively define and influence a new technology’s value proposition. When we quote excerpts from interviews (translated from French to English when applicable), “Dev” refers to technology developers, “Inv” to capital investors, and “Reg” to regulators. Other alphanumerical indexes refer to media excerpts.

## Results

### Entrepreneurs

When health technology-based ventures are created, they often lack business expertise and may not have a clear idea of how their technology will create value. Yet, entrepreneurs must articulate a business plan to obtain resources from capital investors.^11,21^ The entrepreneurial clinical teams we studied gave an initial direction to the technology development process and made, later on, a number of compromises to address clinical, business, and regulatory concerns. These entrepreneurs pursued different motivations such as successfully engineering a technically sophisticated solution, bringing about a clinical paradigm shift or seeking to help patients. For one of the developers we interviewed, a new technology should possess, in principle, at least three valuable features:

*“First, it must fulfill a need patients have. Then, it must be effective, be able to treat not only 40% of patients. And it must be efficient. So we’re talking about productivity too. ‘Effective’ is about doing the right thing, ‘efficiency’ is about doing many more things. So it means treating many patients with lower costs for healthcare systems”* (Dev C1).

Although strong input by clinicians helps to increase the clinical relevance of the value proposition of a new technology, the final technology may not always reflect this input. These entrepreneurs have to strike significant compromises to expedite sales and generate revenues and system-level challenges may easily skip under their radar.

For example, the initial goals of the labour decision-support software venture of engaging women more actively in their care and of reducing unnecessary C-sections vanished. This venture gathered user feedback long after its key R&D activities took place and faced significant commercialization challenges: because “high volume productivity is very lucrative for a physician,” the technology “may be best for the patient, but it is financially not best for the physician. It may be morally and socially good for the physician, but there’s a financial penalty” (Dev L1). Realizing that its value proposition was not sufficiently appealing to obstetricians, the venture decided to target physician insurers. Its design efforts were then geared at providing value to risk liability managers by furthering the medico-legal and administrative features of the system (with the aim of documenting care processes in case things go wrong). Hence, the types of benefit a technology may eventually bring to healthcare systems not only depend upon the users one seeks to satisfy (physicians, nurses, patients, etc), but also upon the likely purchaser (hospitals, physician insurers, national health systems, etc).

We examined in greater depth how business models influence technology design priorities. A business model articulates a technology’s value proposition with its market segment, revenue model, production system, and commercial strategy.^11^ It, thus, “freezes” the value proposition, including to whom value is offered. The heart ablation catheter venture adopted an archetypical business model for ventures operating in the biomedical sciences. By relying on a large network of clinician-investigators across North America and Europe, it conducted clinical studies in several different areas altogether. These medical specialists contributed to develop the knowledge basis required for regulatory approval, but also to the international clinical marketing of a technology that fitted well with reimbursement systems (ie, fee-for-service). As a result, this venture’s growth meant revenues for entrepreneurs, investors, and users.

In sharp contrast, the capacity of the home monitoring system venture to grow and generate revenues was very limited. The initial goal was to create a clinically-oriented system that “could provide those who are really sick with a personal assistant that would help them to follow their health status and share that data with clinical staff” (Dev N2). For achieving this goal, a collaborative approach was established at an early stage:

*“We started getting lots of feedback from healthcare providers. All of us, the 4-5 programmers at that time, we were working in a room the hospital had given to us. The providers developing the clinical protocols for our system were there too. As soon as they had an issue, they would turn to us, and we were right beside!”* (Dev 2N).

Even though effective partnerships with hospitals and a co-design strategy with users enabled the venture to provide value to clinicians and hospitals, its business model remained fragmentary. The monitoring system addressed an important system-level challenge since it was designed to reduce unnecessary hospitalizations and emergency room visits. Such a technology could, in principle, create value for hospitals that have incentives to prevent deterioration of chronically ill patients, such as health maintenance organizations (HMOs) in the United States, or coordinated care models in publicly funded systems. Nevertheless, the venture never really succeeded in articulating a revenue model that could translate the “distributed benefits” it offered (to patients, clinicians, hospitals, homecare organizations, and third-party payers) into a single stream of revenues. The technology was designed as if the patient’s best interest was a sufficient incentive for hospitals and homecare providers to work collaboratively and thus, together “buy in” the new technology. This proved very hard to achieve in practice and the venture was sold before its entrepreneurs could find out ways to scale up its technology.

Overall, for entrepreneurs who tap on their healthcare experience to create a venture, defining a new technology’s value proposition entails a double pledge: their technology shall be able to generate revenues in order to engender health benefits.

### Investors

Investors who decide to support ventures commit their resources for a specified period of time with the aim of recouping their investments and generating returns. They actively transform the value proposition of a new technology since their aim is to bring the venture to the most profitable “exit,” which may happen through the acquisition of the venture by another company or an initial public offering (IPO) that provides the ability to sell shares to the public. To diversify risks and increase the likelihood of success, investors’ decisions are made within the context of a broader investment portfolio and their role is to maximize the shareholders’ good.^10,17^

*“We’re portfolio managers [...]. Over 10 investments we gonna do, in general, there’s one that will bring in 20, 30, 40 times our bet. There’s going to have 2 or 3 that will go bankrupt, and with the other 6, either we’ll lose a little, or we’ll recover a little more money. So basically, for us it’s the average, it’s the batting average: my shareholder will be happy if, say, that with the $ 100M that I’ll put in ... I don’t know in 10-12 investments —these are arbitrary numbers—I bring back to my shareholder 120-130M $. So this is when he’s happy”* (Inv4).

While investors recognize that they rely on “a process that remains very subjective even if it’s dressed up with all kinds of facts” (Inv3), when they choose to support a particular venture they ask entrepreneurs to come up with a business plan in which the opportunities for growth are clear and impressive. How swiftly a venture progresses is of interest not only to investors, but also to business analysts and journalists who comment publicly on its promissory value. Below is a typical business press excerpt wherein the core innovation is described as holding the potential to address multiple clinical needs, which represent large or growing markets:

*“[Breast imaging technology] should be on sale in 2002, launched into a worldwide market for diagnostic imaging systems that topped US$10.1 billion in 1998. Experts predict it will grow to over US$14.7 billion by 2004. [CEO] has plans to scale: ‘We’re already looking at applying this technology to brain cancer and other tissue —the potential is huge’”* (GB-4).

The media emphasis on opportunities for growth is a not a simple form of hype. Investors do not engage in plain business activities, but in business activities that offer *speculative* opportunities. What matters is the value ventures may generate within a period of 5-7 years. These ventures must attract additional investors and/or shareholders. And they must grow.

While the business-oriented media does not address the health value of a new technology in much detail, it provides potential investors and business partners with figures that translate health risks into potentially lucrative markets. For instance, the business case for the labour decision-support software emphasized litigation costs:

*“From the perspective of a physician malpractice insurer, obstetrical liability payments usually form the largest portion of all malpractice dollars paid out. One obstetrical complication, shoulder dystocia, is responsible for anywhere from 900 to 1500 permanently disabled newborns every year and constitutes the second highest category of payouts in OB”* (SNB-1).

While reducing malpractice costs may not be considered a health policy goal per se, such risks do represent a significant business opportunity. In fact, from an investment standpoint, the technology a venture is developing has no intrinsic value: it is neither good nor bad. The issue is whether it can “find its purchaser” and sell.

*“Unfortunately, the quality of the technology and the fact that it is marketed and marketable, sometimes there isn’t necessarily a match between the two. You can have a great technology that will end up forgotten and you can have something that is very ordinary but that will be a commercial success”* (Inv1).

This logic creates tension with clinically trained entrepreneurs who believe their technology possesses clinical value and often struggle to improve its performance within the time and money constraints their investors have set. Investors seek the commercialization path with the least resistance and invariably prioritize compromises that may expedite sales.

Whether the technology delivers its promises (or fails to do so) matters, but it remains, to a certain point, secondary to the value of the venture itself. The fate of most technology-based ventures is to be sold to an established firm when their economic value is the highest. This partly explains why news regarding the boards of directors, partnerships, business awards or financial statements occupies a lot of media space. For speculation to take place, the initial promises of these emerging firms *must* be high. Yet, the fact the technologies will not entirely fulfill their clinical promises is not problematic from the speculative logic of capital investment. To begin with, it is the investment portfolio as a whole that has to generate returns. Then, investors reduce the risks underlying each “deal” by choosing ventures whose value propositions are aimed at large and reachable markets, align with reimbursement systems or do not interfere with physician revenues. Overall, venture capital supports technologies that generate health gains by accident, not by design.

### Regulatory Agencies

Regulatory agencies in North America and notified bodies in Europe set criteria and processes regarding the types of proof health technology-based ventures must produce for pre-market assessment, market clearance or post-market surveillance.^24^ Obtaining regulatory approval is one of the most important business achievements of a venture since it opens up the market, making sales and diffusion within specific countries possible. For regulatory agencies, the value of a new technology lies in its ability to demonstrate its safety and efficacy, ie, that it does what it is supposed to do. Civil servants who execute the appraisals rely on science and must use their expertise and judgment:

*“For a specific device type, we don’t have anywhere written, okay, for this one, you need to have 200, 300, 400, 500 patients enrolled in a trial, before we would allow this to be cleared. I don’t think we have these written out. For some technologies, I guess they know by experience that you’d want to see a clinical study with so many patients because less would not show the outcomes that you’re looking for, you know, the end points of the study. So, I think … it’s kind of absolute that the device has to be safe and effective, but there is … there’re degrees to which there is safety. Depending on what the device does, maybe different numbers or different patients you would need to show, to demonstrate the safety and effectiveness”* (Reg2).

Unlike HTA or coverage with evidence strategies, regulatory approval does not examine costs, clinical relevance or impact on healthcare systems.

*“We sometimes see applications which we call ‘me too’ applications. It’s no different from twenty other ones out there, but the manufacturer wants to get in this business and we say fine. You know the world doesn’t need yet another one of these things, but fine if you want to sell one, you get one. So we don’t judge whether this is really beneficial or new*” (Reg3).

One may, thus, wonder whether the early production of regulatory evidence that coverage with evidence development strategies call for are likely to affect, at an early stage, the innovativeness or types of benefit of the new technologies entrepreneurs are seeking to develop. Yet, one important way in which regulatory agencies partly affect the value proposition of a new technology is by requiring structured information regarding the technology as well as the venture seeking to bring it to market. On the one hand, depending on the level of risk of the device it is developing, the venture has to produce specific types of proof and thus, conduct clinical trials. On the other hand, it must show the soundness of its corporate structure, manufacturing facilities and governance. Regulatory agencies, thus, indirectly bring about structural changes in ventures by asking them to produce evidence and to adhere to various ISO norms.

Regulatory experts operate on scientific grounds that are close to health research, but their decisions are bounded by the mission of their agency, which is to decide whether market access is warranted or not: “*We would not take costs into consideration either at licensing time or post market. It’s sort of... do you want to buy the cheapest car on the market or do you want to buy a fancy car. The cheap one isn’t as nice as the fancy one, but they’re both legal*” (Reg3). This logic reinforces the “double” value of an innovative health technology, as a health intervention, which depends upon its ability to generate health gains and as an economic commodity, which depends upon its ability to generate profits.

Obtaining regulatory approval is important for health and economic reasons altogether since it legitimizes the demand for a new technology: physicians, hospitals, and patients are likely to trust the technology is safe and effective, and investors and shareholders can estimate the size of the markets to be seized. In addition, if safety problems arise once a technology has been approved, established post-marketing procedures will help to protect the public and the manufacturer and its shareholders too. By adhering to these procedures, the ventures can justify their decisions and protect the value of their business. Albeit they do so involuntarily, regulatory agencies provide economic worth to ventures. This is all the more evident for firms that are listed on the stock exchange since market clearance, plus the prospect of sales within large geographical markets, boosts investors’ confidence. Such events figure prominently in the business news with the consequent increase in the share price.

Ultimately, regulatory approval “freezes” the value of a new technology. For instance, a decision to improve an early version of the heart ablation catheter approved by the American Food and Drug Administration (FDA) was made by considering whether or not the current market for this product “pays for a new clinical study” (Dev A1). Estimating that such a study would cost around $10-15 million and that sales would not be dramatically increased, the catheter remained as it was when first approved. In fact, once approved on the market, only minor changes can be brought to a technology’s design and manufacturing process without necessitating additional regulatory review.

To summarize, our findings clarify how entrepreneurs, investors, and regulatory agencies respectively define and influence a new technology’s value proposition. More specifically, Table 3 indicates that value is defined as a double pledge by entrepreneurs, as a speculative financial opportunity by investors and as requiring safety and efficacy evidence as well as organizational auditability by regulators. Each of these definitions of value may be seen as partially complementing each other, but they also generate tensions in the way the value proposition of a new technology is transformed at an early stage: an issue that we discuss in further detail below.

**Table 3:** A Summary of How Entrepreneurs, Investors, and Regulatory Agencies Define and Influence a New Technology’s Value Proposition

	**How Value Is Defined**	**Impact on the Value Proposition**
Entrepreneurs (clinical teams)	Value entails a double pledge: • A profitable business model • Health benefits	Formulation of an initial value proposition that can be developed and tested through clinical studies
Capital investors	Value is speculative: • To generate returns, a technology-based venture must grow within a pre-determined timeframe • Health risks represent a business opportunity	Transformation of the value proposition into a “sellable” product that: • Addresses large and reachable markets • Generates clinical value for physicians and does not conflict with revenues • Aligns with reimbursement systems
Regulatory agencies	Value requires evidence of: • Safety and efficacy of the technology • Auditability of its manufacturer	Production of proofs • Market clearance increases the economic and clinical value of the venture and brings the innovation process to a halt

## Discussion

We now summarize our study’s contribution and provide policy insights regarding the interplay between innovation policy and health policy, which shapes new health technology by deploying different logics and valuing different outcomes.

### Contribution of our Study

We began this paper by underscoring the lack of coordination mechanisms between innovation policy and health policy that typically prevails in industrialised countries.^5^ This may be partly explained by the historical processes by which governance structures gradually took shape since the emergence of the welfare state. Yet, such a lack of coordination proves problematic considering the complexity, policy impacts and costs of today’s health innovation.^25^ Within this perspective, our study’s contribution to current knowledge is, we believe, two-fold.

First, our findings provide a detailed understanding of the way key upstream actors define and influence the value proposition of new technologies. For clinicians who create ventures, value lies in a double pledge wherein clinical improvements and a profitable business may dovetail (see Table 3). They have a direct influence over a new technology’s value proposition by articulating clinical needs and providing precious input regarding the context in which the technology will be used.^2,3,5^ Yet, the fate of their entrepreneurial endeavor remains largely structured by financing strategies and regulatory requirements. Investors value innovations that can find a market — no matter their intrinsic value for clinical practice or healthcare systems. They actively influence a new technology’s value proposition by prioritizing technology design decisions that facilitate sales. Regulatory agencies value proofs of safety and efficacy, but ignore relevance and costs. While their requirements concern a limited part of a new technology’s value proposition, these agencies increase the economic value of the ventures that obtain market approval. This point is important since entrepreneurs and investors generally criticize regulatory agencies for the time they take to do reviews and often characterize regulatory requirements as hindering the innovation process.^6,9,15^ Our study rather shows that those who invest in ventures also extract value from the regulatory process.

Second, our findings lend support to the body of policy-oriented initiatives that seek to interface health policy with innovation policy.^6-9^ Although their respective impact on the value proposition of a new technology appears uneven, both innovation policy and health policy affect the way small firms may conceive of, and develop, innovative technologies. Whereas the value of a new technology for innovation policy primarily lies in the economic activities it may generate, its value for health policy requires evidence regarding the nature and size of its benefits.^14,26,27^ As our findings showed, to secure investments entrepreneurs must make a persuasive business case, but as they progress along the innovation development pathway, it is the regulatory requirements that condition the kinds of evidence they will generate. During this timeframe, entrepreneurs have to make technology design compromises, including some that significantly transform the value of the new technology. For instance, a technology like the decision-support software could be seen as valuable from a health system perspective if it effectively reduced unnecessary C-sections, but less valuable if its main outcome is to reduce risks for medical insurers. Notwithstanding the fact that the value of an innovation may still change once it becomes more widely adopted,^3^ the compromises that investors and entrepreneurs agree upon do affect the type and importance of health system benefits a new technology may deliver. One must also acknowledge that innovation stakeholders do not operate within a similar time horizon to provide value to an innovation. For instance, entrepreneurs focus on improving the technology’s performance for specific clinical indications, whereas investors are aiming for large markets and rapid growth. Hence, policy-oriented initiatives such as early HTA and coverage with evidence development may provide technology developers with precious input regarding the health consequences of the compromises that are considered at an early stage. Yet, to foster technologies that bring more value to health systems, we believe a more comprehensive health innovation policy is necessary.

### Policy Insights

For several policy observers, the 2 policy agendas of “health” and “wealth” can be reconciled.^20^ The presumption is that encouraging entrepreneurial activities in the domestic health technology industry can generate economic and health benefits altogether. For instance, the Canadian Advisory Panel on Healthcare Innovation recommended that Health Canada in collaboration with Industry Canada develop “a whole-of-government federal strategy to support the growth of Canadian commercial enterprises in the healthcare field” and thus, better exploit the “dual potential” of this industry.^28^

Such emphasis is not entirely surprising. The premise that technology-based ventures support economic growth pervades innovation policy frameworks across industrialized countries. Yet, this premise is poorly supported by current scholarship on entrepreneurship. For Nightingale and Coad, innovation policy-makers fail to recognize the limited generalizability of the available evidence:

“*In some atypical places like Silicon Valley, high-tech entrepreneurship can be a major driver of innovation and economic growth, but care must be taken in extrapolating from these exceptional conditions. But in many other areas the evidence suggests the contribution of entrepreneurial start-ups to the economy is limited and in some cases can be potentially damaging.*”^18^

Our findings suggest that supply-side logics shape the kinds of innovation patients and healthcare systems get more powerfully than demand-side logics. Considering that a technology that “sells” may not align with health system level priorities, we do not believe that the tension between health and wealth goals can be resolved without a deliberate policy intervention to foreground demand-side logics. In other words, health policy-makers should actively support the consideration of health policy issues in innovation policy. This should be facilitated by the fact that the literature on the downstream phases of health innovation is abundant, showing the many obstacles and challenges clinicians, managers, and patients face.^1,2^ These challenges may relate, more specifically, to the *diffusion* phase, that is, an innovation’s passive spread, to its *dissemination* phase when “active and planned efforts to persuade target groups” to adopt it are pursued, to its *implementation* phase when similar efforts to mainstream it are deployed within organizations or to its *sustainability* phase when it enters into routine use (until it reaches obsolescence).^25^

What our study has foregrounded is the notion that many of the challenges characterizing the later phases of the innovation lifecycle are determined at a much earlier stage, when the ventures are seeking to develop a new technology as well as a preliminary business plan in order to secure capital investments.^21^ Within this perspective, Box 1 summarizes our fieldwork into five lessons that can inform a more comprehensive health innovation policy. Such policy has to ponder the different logics at play and the kinds of outcome that are valued, measured and rewarded by innovation stakeholders and which barely overlap with the needs and challenges of healthcare systems.

**Box 1. Five Lessons for Health Innovation Policy**

- Clinical leadership and user involvement are not sufficient for a new health technology to respond to important healthcare system needs and challenges.
- The initial value proposition is malleable, but the business model “freezes” this value at an early stage.
- Initial promises must be high and the fact that they rarely will be realized is not problematic from a speculative standpoint.
- Venture capital supports valuable health technologies by accident, not by design.
- Regulatory approval provides both economic and clinical value to health technology-based ventures.

## Limitations and Further Research

To our knowledge, this is the first empirical study to examine 3 categories of actor who profoundly structure how health technology-based ventures emerge. Very rarely scientific journals in the health field publish studies that examine the viewpoints of entrepreneurs and investors on health technology development processes. Such studies are traditionally directed to innovation management and entrepreneurship journals. Considering that these journals cannot do full justice to health policy issues, studies like ours are not without limitations, but bring important insights to a health policy audience.

The use of an explicit framework to synthesize the empirical evidence we examined in greater depth in separate publications increases the credibility of our findings.^22^ Our study brought to the fore meanings and logics that are largely shaped by institutional rules and, as such, may not require a very large sample of respondents to attain empirical saturation. Our study does, indeed, differ from qualitative research that primarily explores perceptions, attitudes or motivations that may span a large spectrum (for instance, attitudes and practices toward childhood vaccination). It should also be underscored that entrepreneurs and investors were eager to share with us their expertise and knowledge, they were passionate about their work and candidly share their views about the innovation development and policy issues they felt required improvements. Indeed, we were not seeking to gather information that had a strategic business or financial value and which could have generated discomfort.

The transferability of our findings to other settings should be appraised acknowledging that innovation policies in Canada are influenced culturally and economically by the United States, but its health policies are aligned with those of European countries where healthcare is publicly funded like the United Kingdom.^13^ In addition, Canadian health technology firms are strongly dependent on exports to North American and European markets and must, therefore, be responsive to the commercialization challenges of an international market. The international nature of health technology development and the important similarities innovation policies share across industrialized countries^17^ suggest that our findings can help to understand health innovation processes in other settings. Nonetheless, our findings focused on ventures and are, thus, not reflective of the R&D activities performed by large medical device manufacturers.

Our findings pinpoint areas for further research. For instance, are there innovation policy instruments that formally require a more active role on the part of demand-side actors? Do such policy instruments prevent cooptation of users and third-party payers by supply-side actors? Further research could examine how supply-side actors respond to the strategic importance of protecting the sustainability of healthcare systems. Garber and colleagues suggest that American policy-makers should “offer greater financial rewards for inventing low-cost technologies—and less reward for inventing high-cost ones.”^14^ Considering that such a technology creation paradigm shift “could benefit patients across the globe,” further research on technological innovation and healthcare system sustainability is warranted.^14^ Similarly, it would be worthwhile to explore how new approaches such as responsible research and innovation (RRI), could be adapted more specifically to health innovation.^29^ RRI, which is gaining traction in the European policy landscape, not only calls for the involvement of multiple stakeholders, including the publics, but it also argues in favour of a deliberate and continuous *ex ante* consideration of what is collectively expected from innovation. Further research could examine how RRI helps to handle the uncertainty that characterises innovation at an early stage by implementing inclusive anticipatory and participatory processes that foster reflexivity and responsiveness.

## Conclusion

Since the late 1980s, industrialized countries have been seeking ways to better manage technological innovation in health while ensuring safety, innovation and access.^12^ For Sapolsky, the result of these contradictory desires is that “there is much more promotion than control of medical progress.”^12^ Our study confirms that health policy scholars should carefully examine innovation policy because established upstream dynamics shape in important ways the kinds of innovation patients and healthcare systems get. One key challenge is to understand how innovation policy can foster the development of technologies that address the most pressing healthcare needs and not those that threaten the sustainability of healthcare systems.

## Acknowledgments

This paper greatly benefitted from the constructivism criticisms of the Editor and six anonymous reviewers. We would like to thank the participants of this study who graciously accepted to be interviewed. Myriam Hivon, Bryn Williams-Jones, David Urbach, and Christopher Longo were involved in the broader research program from which this paper draws its data. This research was funded by an operating grant from the Canadian Institutes of Health Research, Ottawa, ON, Canada (CIHR; #MOP-89776). During this 5-year study, the first author held a Canada Research Chair on Health Innovations (2005-2015). The fourth author holds a Canada Research Chair on Health Care Governance and Transformation. Our research group infrastructure is supported by the *Fonds de la recherche en santé du Québec* (FRQS).

## Ethical issues

Ethics approval was obtained from the Faculty of Medicine of University of Montreal, Montréal, QC, Canada (#328-CERFM).

## Competing interests

Authors declare that they have no competing interests.

## Authors’ contributions

All coauthors have made a substantial contribution to the manuscript; they revised it critically for important content and approved the final version.

## Authors’ affiliations

^1^Department of Health Management, Evaluation and Policy, School of Public Health, University of Montreal, Montréal, QC, Canada. ^2^Institute of Public Health Research of University of Montreal (IRSPUM), Montréal, QC, Canada. ^3^University of Montreal Chair on Responsible Innovation in Health Montreal, Montréal, QC, Canada. ^4^Institute of Health Policy, Management and Evaluation, University of Toronto, Toronto, ON, Canada. ^5^École Nationale d’administration publique (ENAP), Quebec City, QC, Canada. ^6^Department of Management, Faculty of Social Science & Public Policy, King’s College, London, UK.

## Key messages

**Implications for policy makers**

- Entrepreneurs, investors, and regulatory agencies influence a new technology’s value proposition.
- Entrepreneurs have to strike compromises to expedite sales and generate revenues.
- Investors support technologies that generate health gains by accident, not by design.
- Albeit they do so involuntarily, regulatory agencies provide economic worth to ventures.
- Health policy scholars should contribute to innovation policy.

**Implications for public**

The value a new health technology may ultimately bring to healthcare systems is defined at an early stage. This value is shaped by the entrepreneurs who create a new company to design and commercialize the technology, by those who invest in their venture and by regulatory agencies. Even when entrepreneurs possess formal clinical training, they strike significant compromises to expedite sales. Health policy issues should be considered by innovation policy-makers because investors support technologies that generate health gains by accident, not by design.
